# More Than Skin Deep: A Case of Nevus Sebaceous Associated With Basal Cell Carcinoma Transformation

**DOI:** 10.7759/cureus.9386

**Published:** 2020-07-25

**Authors:** Shauna Maty, Kristen Salana, Mihaela Radu, Cristina Beiu, Robert Hage

**Affiliations:** 1 Dermatology, St. George's University School of Medicine, St. George, GRD; 2 Dermatology, Emergency Clinical Hospital "Sf. Apostol Andrei", Constanta, ROU; 3 Oncologic Dermatology, Elias Emergency University Hospital, "Carol Davila" University of Medicine and Pharmacy, Bucharest, ROU; 4 Otolaryngology, St. George's University School of Medicine, St. George, GRD

**Keywords:** nevus sebaceous, basal cell carcinoma, gene mutations, mosaicism, dermatology

## Abstract

Nevus sebaceous is a congenital epidermal lesion that typically presents in infancy from the neck up and rarely undergoes malignant transformation. In patients who do present with malignancy, both RAS oncogene and PTCH tumor suppressor gene mutations have been implicated. We report an unusual case of nevus sebaceous in a 41-year-old male patient that developed into basal cell carcinoma on the forehead, and elaborate on the prolonged nature and unique location of its presentation. The case highlights the need for early intervention and how variable access to primary care can impact patient outcomes. We further explore the role of gene mutations in the circumstance that nevus sebaceous does become malignant, as well as pertinent differential diagnoses to consider.

## Introduction

Nevus sebaceous is a type of rare congenital birthmark or skin hamartoma found in up to 0.3% of neonates that typically present from the neck up, most commonly found on the scalp. They are well-circumscribed, smooth, hairless “plaques” composed of sebaceous glands that are benign and do not usually cause any issues other than cosmetic concerns [1,2]. Known to change throughout adolescence under possible hormonal influence and increased activity of sebaceous glands, nevus sebaceous presents in adulthood with a thickened epidermis and has a more bumpy or scaly appearance [2]. Most common presentation is a single plaque on the scalp. In this case study, we report on a 41-year-old male who presents with evaluation of a large multiplaque nevus sebaceous extending from the hairline distally down toward the right eyebrow that has been associated with recent cutaneous changes and possible malignancy.

## Case presentation

**Figure 1:**
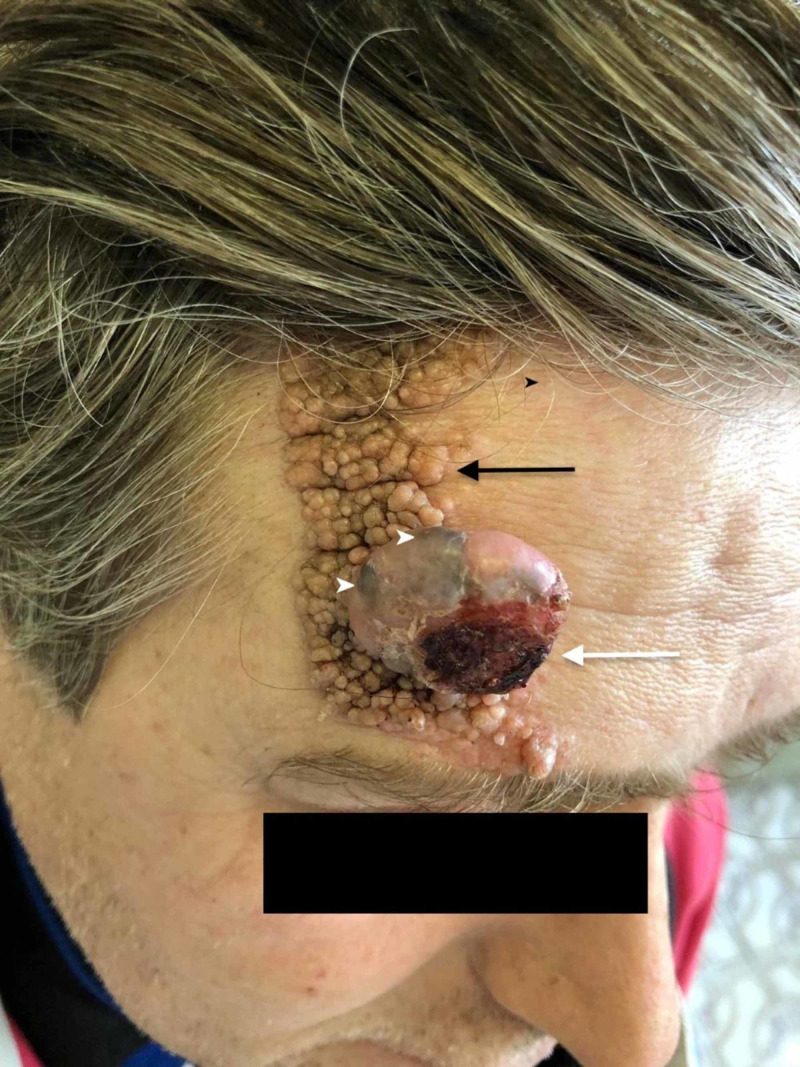
Clinical aspects of the lesion Clinical picture showing a centrally ulcerated pink nodule (white arrow), which developed from a sebaceous nevus (black arrow). The arrowheads point to the areas of dark pigmentation. The blue-black areas are pigmented as such due to melanin production within the tumor.

**Figure 2:**
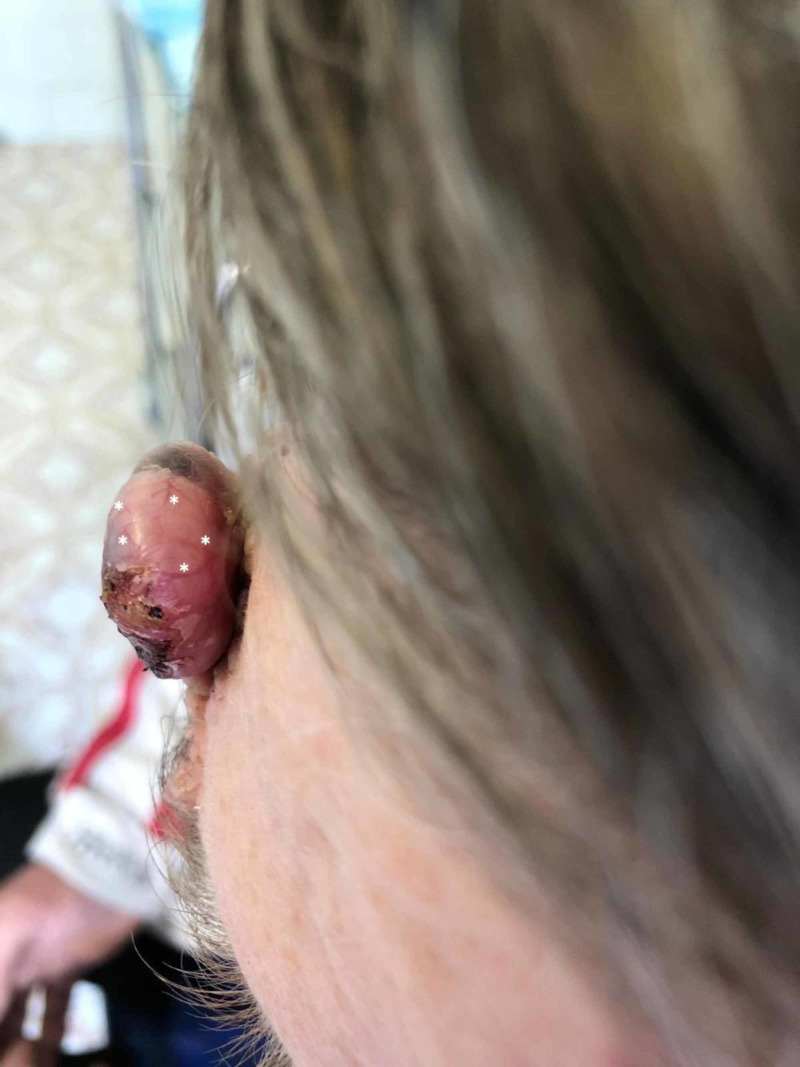
Top-down view of the tumor A top-down view of the tumor, highlighting multiple telangiectasic arborized vessels (white asterisk).

A 41-year-old Caucasian male presented for evaluation of a large multiplaque nevus sebaceous on his scalp and forehead that had recently begun significantly changing. It was reported that the original nevus sebaceous had been present since birth. Three years ago, a pink nodule had emerged from the nevus sebaceous approximately mid-way between the hairline and the right eyebrow. The patient reported that within the last two months this nodule had begun gradually enlarging and beginning to bleed, prompting his decision to seek care. Physical examination revealed a pink nodule with an average diameter of 4 cm emerging from the nevus sebaceous with arborizing vessels, areas of darker pigmentation, and a central ulceration partially covered by a hemorrhagic crust (Figures 1, 2). 

**Figure 3:**
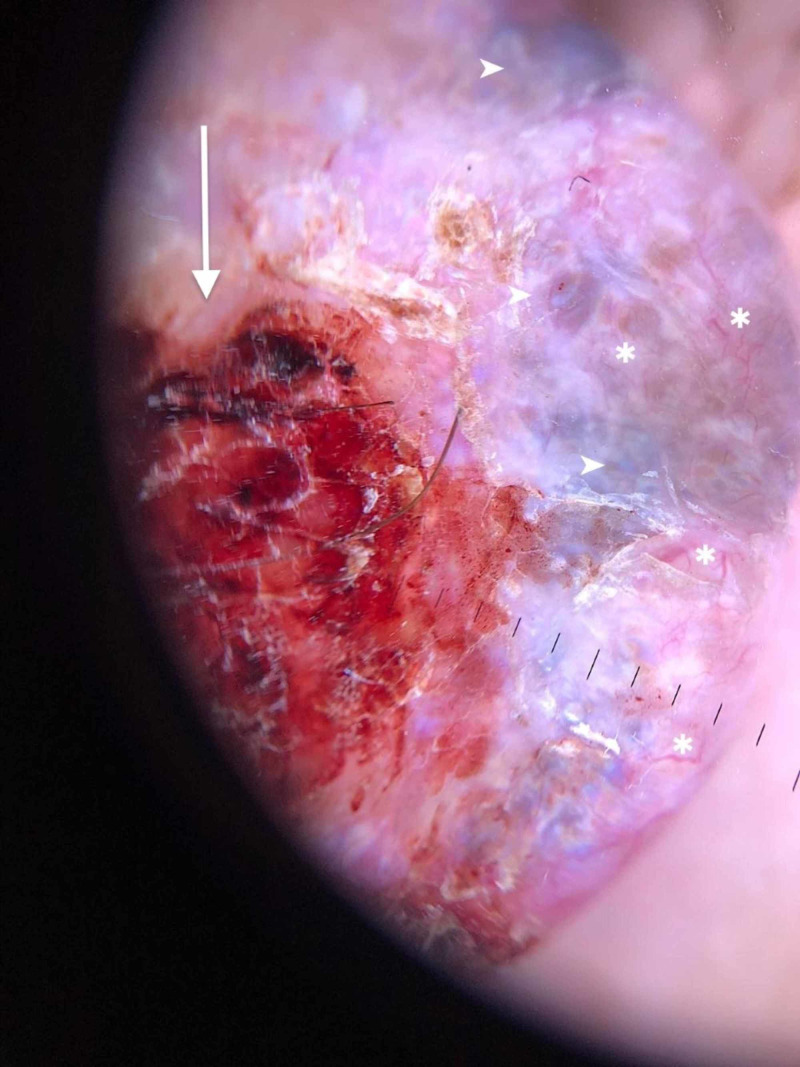
Dermascopic evaluation of the lesion Dermoscopic picture showing multiple blue-gray ovoid structures (white arrowheads), multiple branched telangiectasias (white asterisk), and a central structureless area of ulceration (white arrow).

Dermoscopic evaluation revealed that the pigmented area contained multiple blue-gray ovoid structures of various sizes, both grouped and unconnected, as well as some branched telangiectasias. These characteristics strongly suggest basal cell carcinoma (BCC) (Figure 3). An excision biopsy was then performed and histologic findings confirmed the diagnosis of nodular pigmented BCC. 

## Discussion

The patient was an outdoor worker on a farm in rural Romania, and subsequently reported high chronic sun exposure with minimal protection for the majority of his life. The patient did not report any other health concerns or pertinent family history. After confirming BCC within the nodule via biopsy, the lesion was surgically excised. Unfortunately, the patient was lost to follow-up after surgical excision.

Prior to becoming a member of the EU in 2007, the austerity policies enforced by communist leader Nikolae Ceaușescu drastically reduced public healthcare availability in Romania [3]. As the patient was born in 1972, national impoverishment and minimal public health spending may have prevented early diagnosis in infancy and continuous monitoring of his condition throughout his lifetime. It is notable to mention that although 85% of Romanians have access to comprehensive health coverage, healthcare expenditure in Romania is less than any other nation in the EU [4]. One of the major inequalities that exists within Romania is the unequal distribution of medical providers and limited access to healthcare for the rural population. As of 2015, up to 9.4% of Romanians reported that they had received inadequate medical care due to cost as well as the distance from their nearest medical provider.

Upon initial clinical evaluation of a suspected nevus sebaceous, differential diagnoses are important to consider as similar lesions exist. During infant examinations, it is important to consider the appearance of cutis aplasia, solitary mastocytomas, and congenital triangular alopecia as they can present similarly to nevus sebaceous [5]. Further, nevus syringocystadenomatous papilliferus (SCAP) lesions can look very similar during clinical examination, but often have a more nodular, pink surface rather than the yellow, smooth plaque of nevus sebaceous. It has been reported that SCAP lesions can be associated with nevus sebaceous in up to 33% of cases, and associated with BCC in 10% of cases [6]. Giant congenital nevus was also considered as a differential; however, they tend to appear much darker due to the excessive growth of melanocytes which was not present in the patient, and tend to be both hairy and stay within the hairline [7]. Finally, a growth known as linear sebaceous syndrome or Schimmelpenning-Feuerstein-Mims syndrome is important to consider as it is characteristically associated with a large, linear nevus sebaceous. However, this kind of growth tends to be highly correlated with systemic abnormalities, even affecting the central nervous system, which is not seen in this case [7]. Once the differential diagnoses were considered and ruled out, a diagnosis of nevus sebaceous was made (Table 1).

**Table 1:** Summary of differential diagnoses related to nevus sebaceous

Differential Diagnosis	Pertinent Positives	Pertinent Negatives
Cutis aplasia	Present in early infancy as focal erosion of scalp [8]	Smoother papyraceous surface; healing with atrophy and scarring [8]
Solitary mastocytomas	Present in early infancy [8]	Pink, nodular surface; favors distal extremities [8]
Congenital triangular alopecia	Congenital; localized to frontotemporal scalp [8]	Triangular temporal hair loss; size of lesion unchanged through lifespan [8]
Syringocystadenomatous papilliferus (SCAP)	Association with nevus sebaceous; yellow papules; lesions increase in size at puberty [9]	Presents in association with apocrine adenoma, trichoblastoma, and eccrine poroma [9]
Giant congenital nevus	Present in early infancy [8]	Tan color; favors extremities and trunk; hairy lesion [8]
Schimmelpenning-Feuerstein-Mims syndrome	Epidermal nevus present in early infancy [8]	Seizures and intellectual disability; multisystem manifestations [8]

Nevus sebaceous has been diagnosed under several names, including nevus sebaceous of Jadassohn, organoid nevus, and Jadassohn Disease II [2]. Recent genome sequencing of affected patients has revealed the role of mosaicism in the pathogenesis of nevus sebaceous through HRAS and KRAS mutations [10]. In the patients studied, 95% shared a HRAS mutation and 5% shared a KRAS mutation in lesion tissue biopsied, while non-lesional tissue displayed a wild-type sequence. In particular, HRAS p.G13R was determined to be a hotspot mutation, which may increase the risk for the secondary development of malignancy in individuals with this substitution [11]. RAS genes promote cell growth, and activating RAS mutations have been strongly linked to the increased cancer risk and hamartomatous growth [12].

BCC arising from sebaceous nevus has been reported in only 1.1% of cases and is even argued to be as low as 0.8% [1,13]. However, the conditions do share a deletion of the PTCH tumor suppressor gene on chromosome 9q22.3 [14]. Malignant transformation more commonly occurs in patients within the age range of 40-70 years [15]. A study done by Cribier et al. microscopically evaluated 596 cases of nevus sebaceous [16]. They reported that only 0.8% of cases were found to be malignant, and as many as 49.8% of cases were found on the scalp, making the case report of our patient exceedingly rare both in location and progression. In addition, an 18-year review study including 651 patients with nevus sebaceous found only 5 (0.8%) had evidence of BCC, while 10.3% presented with sebaceous nevus on the forehead (most common was the scalp, at 62.8%) [14]. Interestingly, the mean age of those presenting with BCC was 12.5 years, with a range from 9.7 to 17.4 years.

In accordance with the studies evaluated, the common method of treatment for nevus sebaceous presenting with BCC is surgical excision. BCC has a low rate of metastasis, and it is the most common skin malignancy in the Caucasian population [17]. Unlike melanoma, which is the most aggressive form of skin cancer, metastasis rates of BCC range from 0.0028% to 0.55%; however, when it does occur it tends to behave in an aggressive manner with recurrence commonly reported after surgical excision in patients affected [18,19]. Once a diagnosis of BCC is made, early intervention is key to promoting a positive patient outcome [20].

## Conclusions

This case study in particular is valuable to learn from in terms of the patient’s unique forehead presentation extending beyond the hairline, elongated timeline, and seeming lack of any genetic risk factors or comorbidities. High-quality dermoscopy was integral to the diagnosis of the patient in a non-invasive manner. It is important to consider patient circumstances as well in regards to nevus sebaceous: while frequent checkups may be ideal to evaluate and assess the lesion, that is not always realistic or possible. Although the BCC has been successfully removed in this patient, surgical follow-up would be needed for any additional information. The patient was lost to follow-up; thus, it remains unknown whether surgical intervention alone would have been a successful method of treatment for the BCC. Unequal access to primary and specialty care in his rural setting may have contributed to the prevention of early intervention and resolution. 
